# Electromagnetic levitation containerless processing of metallic materials in microgravity: thermophysical properties

**DOI:** 10.1038/s41526-023-00281-4

**Published:** 2023-05-02

**Authors:** M. Mohr, Y. Dong, G. P. Bracker, R. W. Hyers, D. M. Matson, R. Zboray, R. Frison, A. Dommann, A. Neels, X. Xiao, J. Brillo, R. Busch, R. Novakovic, P. Srirangam, H.-J. Fecht

**Affiliations:** 1 Institute of Functional Nanosystems, Ulm University, Ulm, Germany; 2 Department of Mechanical and Industrial Engineering, University of Massachusetts, Amherst, MA USA; 3 Department of Mechanical Engineering, Tufts University, Medford, MA USA; 4 Center for X-ray Analytics, Empa Swiss Federal Laboratories for Materials Science and Technology, Dübendorf, Switzerland; 5 Institut für Materialphysik im Weltraum, Deutsches Zentrum für Luft- und Raumfahrt (DLR), Köln, Germany; 6 Lehrstuhl für Metallische Werkstoffe, Saarland University, Saarbrücken, Germany; 7 National Research Council (CNR-ICMATE), Via de Marini, 6, 16149 Genoa, Italy; 8 Warwick Manufacturing Group, University of Warwick, Coventry, UK; 9 Present Address: Institute of Quantum Technologies, German Aerospace Center (DLR), Wilhelm-Runge-Straße 10, 89081 Ulm, Germany

**Keywords:** Theory and computation, Techniques and instrumentation, Structural materials, Materials for energy and catalysis, Fluid dynamics

## Abstract

Transitions from the liquid to the solid state of matter are omnipresent. They form a crucial step in the industrial solidification of metallic alloy melts and are greatly influenced by the thermophysical properties of the melt. Knowledge of the thermophysical properties of liquid metallic alloys is necessary in order to gain a tight control over the solidification pathway, and over the obtained material structure of the solid. Measurements of thermophysical properties on ground are often difficult, or even impossible, since liquids are strongly influenced by earth’s gravity. Another problem is the reactivity of melts with container materials, especially at high temperature. Finally, deep undercooling, necessary to understand nucleus formation and equilibrium as well as non-equilibrium solidification, can only be achieved in a containerless environment. Containerless experiments in microgravity allow precise benchmark measurements of thermophysical properties. The electromagnetic levitator ISS-EML on the International Space Station (ISS) offers perfect conditions for such experiments. This way, data for process simulations is obtained, and a deeper understanding of nucleation, crystal growth, microstructural evolution, and other details of the transformation from liquid to solid can be gained. Here, we address the scientific questions in detail, show highlights of recent achievements, and give an outlook on future work.

## Introduction

For a long time, the materials scientist’s efforts to produce new materials with desired properties were focused on the solid state of the material, its microstructure, and the resulting physical properties. In the last decade, the largely empirical approach for obtaining a desired microstructure during manufacturing of metallic and semiconductor parts was replaced by a new approach, based on computer simulations of the solidification process^1,2^. Nearly all industrial metal production processes, such as casting, joining, welding, thermal spraying, gas atomization, and single crystal growth, involve melting and solidification of the material. Numerical simulations to guide process development and optimization have become standard tools in industrial fabrication. They offer cost-efficient means to reduce development times to obtain optimized microstructures and high-quality products^3^. The benefits of precise process simulations are an optimized processing route and microstructure, improved product quality, as well as reduced waste production and energy consumption. This is in line with global efforts to reduce greenhouse gas emissions and to achieve sustainable development^4–6^.

However, the simulation of the solidification of a molten metallic alloy is challenging since it involves a complex interplay of physics and chemistry on different length scales, from the atomic scale up to the macroscopic scale. Furthermore, the precise knowledge of the thermophysical properties of the solid and liquid phases are essential prerequisites for meaningful process simulations^7^. In addition to measuring the relevant data for every alloy of interest, accurate model predicted property values could be used to compensate for missing data. But also for the validation of theoretical models, precise benchmark measurements are necessary^2^.

The requirements for precision and accuracy of the measured properties depend on the application for which the properties are used. Sometimes, particularly, for intermediate results like a heat transfer coefficient, theoretical or empirical correlations allow the dependence of properties to be estimated explicitly. For cases where the functional dependence is known, propagation of uncertainties can be calculated using the formalism of GUM^8^.

For example, many correlations for heat transfer in forced convection relate the Nusselt number *Nu* to the Reynolds and Prandtl numbers *Re* and *Pr* according to the equation below. Expansion of the dimensionless groups gives the explicit dependence of heat transfer coefficient *h* on thermal conductivity *k*, density *ρ*, viscosity *µ*, and specific heat *C*_p_. Thus, an uncertainty of 10% in *k* results in a 6% uncertainty in *h*, while *h* is less sensitive to *µ* and *C*_p_.

1 $${Nu}=C\,\cdot\, {{Re}}^{{\,}^4/{\,}_5}\,\cdot\, {Pr }^{{\,}^1/{\,}_3}$$

2 $$h\propto {k}^{{\,}^2/{\,}_3}{L}^{{\,}^{-1}/{\,}_5}{\rho }^{{\,}^4/{\,}_5}{u}^{{\,}^4/{\,}_5}{\mu }^{{\,}^{-7}/{\,}_{15}}{C}_{\text{p}}^{{\,}^1/{\,}_3}$$

For more complicated phenomena like directional solidification, more complicated models are needed. In these cases, the model itself can give the sensitivity to the input parameters by a parametric study. For example, Yang, et al.^9^, found that the primary dendrite arm spacing predicted for CMSX-4 was most sensitive to density and specific heat, among properties studied. The segregation range showed greatest sensitivity to density and thermal diffusivity in the solid. For these more complex models, the propagation of uncertainty also tightens the requirements for the input parameters. This principle is examined in a more general context in^10,11^. The relevant properties of the solid phases can typically be measured by conventional equipment. In the case of liquid metals, the high chemical reactivity hampers their investigation drastically, as reactions between the melt and its container lead to melt pollution. At high temperature, the reactivity is additionally increased. Among others, this is one reason, why the liquid state still remains the least understood state of matter^1,2,12^. Investigation of thermophysical properties of molten metals and semiconductors is hence increasing the general understanding of the liquid state of metals and semiconductors. In addition, it also adds knowledge to the materials technology and engineering efforts on earth. The high chemical reactivity of liquid metallic and semiconducting melts requires non-contact measurement methods using non-conventional, containerless process techniques. To this end, several containerless levitation methods^13–18^, such as the electromagnetic levitation (EML) technique^19–25^ have been developed.

### Containerless processing

In electromagnetic levitation, the electrically conducting sample (typically 1.0 g) is exposed to a spatially inhomogeneous alternating electromagnetic field produced by a water-cooled pair of coils (200 A, 300 kHz). Due to induced eddy currents, the sample experiences a force into the direction of the smallest field strength and it is positioned against gravity, i.e. it levitates. In addition, the sample is inductively heated and finally melts. Electromagnetic levitation is intrinsically stable, as there is a back-driving force for dislocation from the equilibrium position. Thus, even strongly evaporating metals can be processed; the technique is also robust against material pollutions, as long as the electrical conductivity is sufficient. Levitation methods in general yield access to high temperatures, deep undercoolings, and chemically highly reactive materials.

In order to overcome gravity, the electro-magnetic field is comparatively strong which leads to corresponding side-effects^23^, such as turbulent flow^26^ inside the sample. This flow leads to spontaneous excited droplet oscillation- and rotational modes, pronounced translational motion, and also to a deviation of the sample shape from sphericity. Due to the flow, the temperature- and compositional distribution is fairly homogeneous, but the investigation of some important thermophysical properties is prevented, such as the viscosity^27^. In ground-based electromagnetic levitation, heating and positioning are not decoupled and the sample needs to be cooled by admitting a flow of the processing gas to sample, in order to adjust the temperature. Thus, as another limitation, ground based electromagnetic experiments cannot be done under vacuum conditions.

Electrostatic levitation (ESL)^18,28,29^ is aiming to overcome some of these challenges. In electrostatic levitation, an electrically charged sample is positioned against gravity by an electrostatic force generated by an electric field between two electrodes. Heating is accomplished by a one or several IR-lasers pointing at the sample. Thus, heating and positioning are decoupled and a turbulent fluid flow is also not induced. Moreover, the sample is almost spherical, provided that it is sufficiently small. In ESL, processing can be done under vacuum or, optionally, under atmosphere (He, Ar, N_2_, air,).

The technique is generally available for all classes of materials, metals, semi-conductors, ionic liquids/liquid salts, ionic solutions and polar liquids. Also, there is nearly no principal restriction with regards to measurable properties. These advantages demonstrate the high potential of the electrostatic levitation method and justify the steadily increasing number of groups using this technique^18^.

Nevertheless, also electrostatic levitation exhibits some shortcomings. Unlike electromagnetic levitation, the method is not intrinsically stable. High technical effort is thus needed to control the position of the sample and to adjust the fields correspondingly. Due to the absence of turbulence, oscillations need to be excited, and also the sample is often not fully homogeneous with respect to temperature^30^.

Charge loss can also be compensated by thermionic emission^18^. Thus, electrostatic levitation works best for materials with a small work function, e.g. refractories or Zr-based alloys.

For these reasons, electromagnetic levitation^19–24^ is currently the most important levitation technique^21,31^ for electrically conducting metallic samples and the vast majority of commercial alloys, which are prone to evaporation or naturally contain “impurities” like Oxygen, Sulphur, Magnesium, Manganese, Carbon, and others.

In general, ground based levitation experiments are faced with the common challenge that the sample is not force-free. Even if the positional forces compensate the gravitational forces, gravitation still acts on the sample. For instance, the droplet is still subject to buoyancy driven convection and gravitation may also lead to the observed deformations of the sample shape.

Processing under microgravity conditions clearly avoids most of the difficulties that occur in ground-based levitation experiments. In electromagnetic levitation, it allows a de-coupling of heating and positioning. The weak positioning forces in microgravity lead to a reduced fluid flow in the sample, thus enabling measurements under purely laminar flow conditions. The almost perfect sphericity of the droplets significantly improves the precision of the measurement of thermophysical properties. The access to long microgravity times (>5 min), which is possible on board the International Space Station (ISS), is required for measurements in thermodynamic equilibrium, such as the alternating-current (AC) calorimetry method^32^. In addition to the long durations of microgravity, processing under vacuum conditions is also enabled. Electromagnetic levitators on parabolic flights and on ground are typically restricted to certain noble-gas atmospheres, in order to achieve a sufficient cooling rate. The longer process durations, as well as the better microgravity quality on the ISS clearly improves the measurement precision^23,31^. Consequently, the long-duration microgravity environment on board the ISS allows materials science investigations that cannot be done otherwise. Electromagnetic levitation experiments on board the ISS are possible with the electromagnetic levitator (ISS-EML), located in the European science module “Columbus”. For details on the facility, see the overview article in this issue, or Ref. ^22,33,34^

For the sake of completeness, it must be mentioned that since a few years, an “Electrostatic Levitation Furnace (ELF)” is also available on board the ISS^35–37^. This specific device can operate in air or under a nitrogen atmosphere and is mainly designed to investigate inert materials, i.e noble metals or oxides, ionic liquids/salts, and molten ceramics. As these materials cannot be processed in EML, both facilities complement each other in an optimum way.

### Scientific questions

Any improvement of numerical simulations of casting and solidification processes of metallic alloys would lead to an increase of production efficiency, reduction of waste and energy consumption, and an improvement of the product quality and performance. Hence, efforts to increase the knowledge about the thermophysical properties of liquid metallic melts will lead to a benefit for the industrial manufacturing of metal products.

Material classes of interest are therefore:

- structural materials (e.g. steel and aluminium alloys)
- high-performance materials (e.g. Ni-, Co-, Fe-based superalloys)
- functional materials (e.g. semiconductors, such as Si, Ge, etc., magnetic materials, such as Nd-Fe-B, Fe-Si)
- novel materials (e.g. bulk metallic glasses, high-entropy alloys)
- semiconductors

A number of scientific questions have been identified and are addressed by the experiments conducted using the ISS-EML facility on board the ISS. Additionally, a number of future emerging topics were identified which can benefit from the scientific return of the EML experiments in the future.

### Thermophysical properties

The continued interest in the thermophysical property data of metallic melts is a result of the experimental difficulties arising due to high temperatures and gravitational forces during measurement. Some relevant properties (especially for non-reactive melts of low-melting materials, as well as in the solid phase) can be measured on the ground. But for highly reactive metals, having a high melting point, containerless measurement approaches are required to obtain high-precision data of their thermophysical properties^25,38^.

A set of methods to measure thermophysical properties of liquid droplets by electromagnetic levitation was established over the past years and are now used routinely. A compact overview can be found in Refs. ^23,25,27,31,38,39^. These methods allow the direct measurement of the following surface and volume-dependent thermophysical properties:

- Surface tension – using the oscillating drop method^40^
- Viscosity – using the oscillating drop method^40^
- Mass density – optically and inductively^25,41,42^
- Specific heat, thermal conductivity, and total hemispherical emissivity – using AC calorimetry^32^
- Electrical resistivity – using measurements of the samples coupling to the electromagnetic field^41^

These abovementioned thermophysical properties can be used to describe dimensionless numbers that are typically used to describe fluid flow in liquids, such as the Peclét, Prandtl, Rayleigh and Marangoni numbers, that are typically used to describe fluid flow in liquids.

In general, it is also important to measure thermophysical properties of the undercooled melt. As due to possible evaporation, the temperature is limited on the upper end, undercooling simply allows to increase the overall temperature interval by extending the range towards the lower end. Typically for a liquid, no difference is evident in the data between the temperatures *T* above and below the melting point *T*_L_, i.e. the properties obey the same law for *T* > *T*_L_ and *T* < *T*_L_.

But there are some rare but interesting exceptions, where the behavior of the melt becomes different in the undercooled range. For instance, a number of materials show a tendency to de-mix at very low temperature in the sense that homo-coordination becomes preferred. This may become evident in the data of the specific electrical resistivity, but also in the thermal expansion coefficient. Recent examples are Cu-Ni^43^, Si-Ge^44^, or ZrNi^41^ where, at deep undercoolings, a significant deviation of the specific resistivity from linearity is observed or even a sign reversal of the temperature coefficient. Moreover, from investigating the undercooled range, indications of a liquid-liquid phase transition became evident in a number of properties in liquid Vit1 and Vit106a^45,46^.

### Crystal nucleation and glass formation

When cooling a liquid below its equilibrium melting temperature, crystallite nucleation and its subsequent growth are often the first stages of solidification. These processes are intimately related to homogeneous- and heterogeneous nucleation as well as constitutional undercooling. In order to understand these phenomena, investigations of the undercooled melt are required. In addition, non-equilibrium solidification, plays a role in technical processes, where a melt is rapidly cooled and solidified, such as laser powder bed fusion, for example, in the 3D-printing process^47^. Another reason to investigate undercooled melts is the formation of metastable phases which are important for the phase diagram calculations and which are otherwise not accessible. The present understanding of crystal nucleation and glass formation is still incomplete. The classical theory describes the steady-state nucleation rate $J(T)$ as^48^

3 $$J\left(T\right)=\frac{A}{\eta (T)}{\rm{exp }}\left(-\frac{16\pi {\sigma }^{3}}{3{k}_{\text{B}}\,T\,{\Delta {G}_{\text{V}}^{\text{lx}}(T)}^{2}}\right)$$

where $\eta (T)$ is the viscosity, $\sigma$ the interface energy between the liquid and the nucleated crystalline solid phase, and $\Delta {G}_{\text{V}}^{\text{lx}}$ the difference between the Gibbs free energy in the undercooled liquid and nucleated crystalline phase. It is important to stress that Eq. (3) holds for homogeneous nucleation which is linked to heterogeneous nucleation, taking place in equilibrium solidification, via a so-called catalytic factor taking into account the effect of heterogeneous nucleation sites. So, in order to understand heterogeneous nucleation, homogeneous nucleation must be understood first and undercooling promotes its driving force. Currently, the main issue that limits the understanding comes from the lack of precise values of thermophysical properties for the kinetic and thermodynamic contributions. The thermophysical properties entering Eq. 3 are unknown for most alloys of interest, especially in the relevant, i.e. undercooled temperature range. If the liquid is cooled fast enough, nucleation may be prevented, or the growth of the nuclei is very sluggish, and a metallic glass is formed at the glass-transition temperature. Before that happens, the dynamics in the melt freezes in and the study of the process of this dynamical freezing reveals interesting insights into the general physics of collective systems^49^.

Besides complex multicomponent metallic glass forming alloys based on Zr^50^, Fe^51^, or based on noble-metals^52^, also simple binary and ternary compositions were investigated^53–55^.

### Magnetohydrodynamics

Accurate simulations of solidification processes, such as casting, joining, single crystal growth, and additive manufacturing, involving the presence of the liquid phase, require appropriate heat and fluid transport models. The combined effects of fluid flow and electromagnetic coupling have to be considered for some industrial processes, such as cold crucible induction melting or continuous casting with electromagnetic stirring. For this purpose, the magnetohydrodynamics simulations based on the thermophysical properties of the melts, that can be measured using the ISS-EML, are needed. Model validations are possible when quiescent conditions, such as in microgravity, are given, where convection effects are eliminated.

Magnetohydrodynamic models support a wide variety of experiments in this ISS-EML and other facilities which require the flow within the drop to be quantified and qualitatively described. However, the flow is exceptionally difficult to observe in situ during contactless processing. Various techniques including electromagnetic sensors and particle image velocimetry cannot be applied to the molten metallic samples used in electromagnetic levitation. The high temperatures and highly reactive nature of molten metals prevent the use of contact sensors. Meanwhile, the melt is featureless and opaque, rendering imaging techniques ineffectual. Furthermore, when particles are present on the surface of the sample, they are sept into the stagnation lines of the flow and do not provide quantifiable data on the fluid flow^56^. However, the flow may be calculated as a function of the thermophysical properties of the melt, sample geometry, coil geometry, and applied currents through the levitation circuit.

**Fig. 1:**
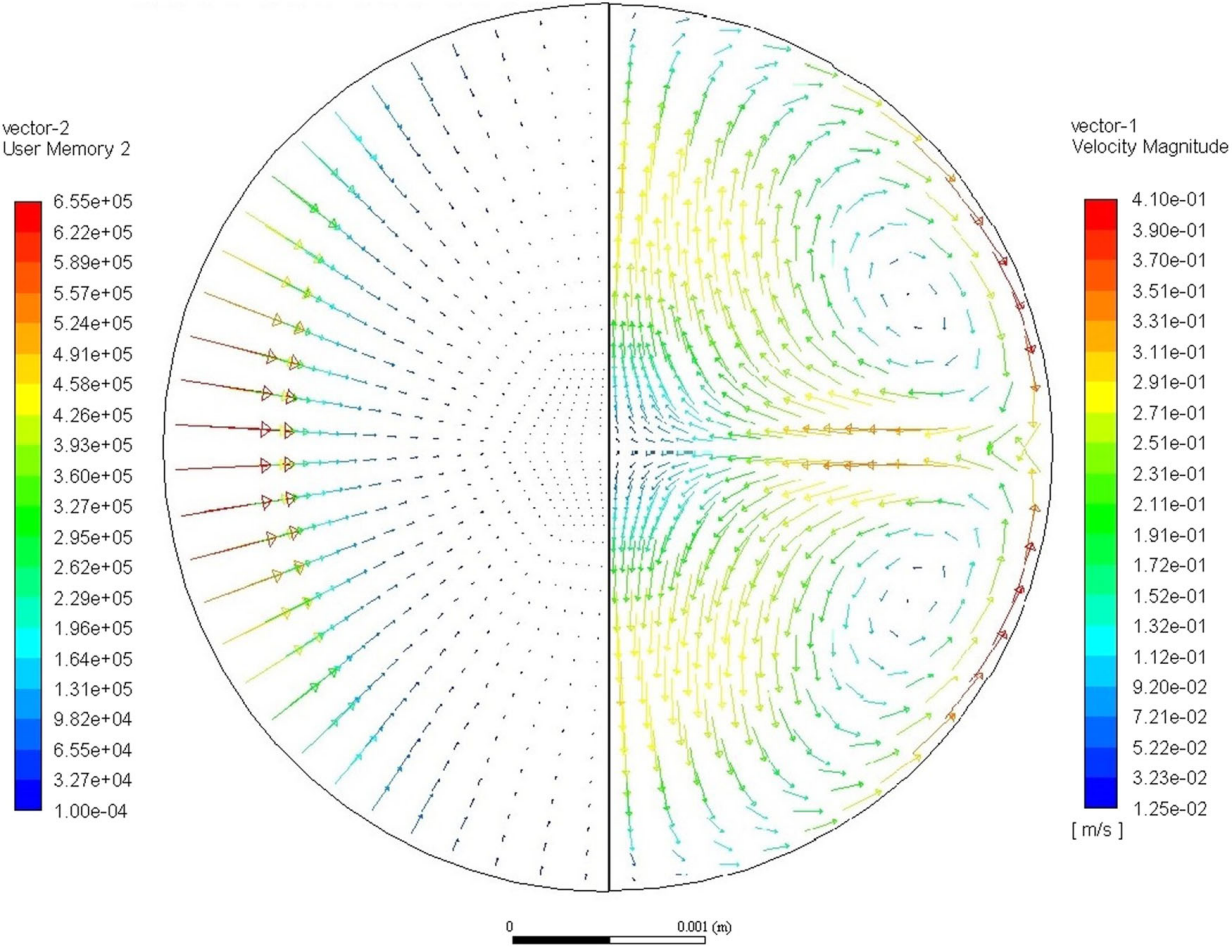
The induced forces, and the flow pattern in the sample. The induced forces are shown in the left hemisphere of plotted sample. The forces shown demonstrate a heater-dominated magnetic field in which the largest forces are applied to the sample along its equator. The flow pattern given in the right hemisphere shows the heater-dominated flow pattern which results from the forces given on the left. This flow pattern features 2 recirculating loops in which the melt is driven into the interior of the sample at the equator and returns to the surface at the poles of the sample.

In the so called SUPOS levitation system, described in detail by Lohoefer and Piller^57^, the sample is positioned by a quadrupole field while a dipole field is used to excite surface oscillations and heat the sample. The forces applied to the sample by the magnetic field are calculated using the conductivity of the melt, sample geometry, coil geometry, and currents applied to the system. A numerical approach, described by Zong, Szekely, and Schwartz^58^, is used to determine the distribution of the magnetic field and the induced currents which in turn allows the Lorenz forces and Joule heating to be calculated for the sample. An example of the resulting magnitude and distribution of the forces applied to the sample is plotted on the left-hand side of Fig. 1.

The magnetic forces are then applied to a computational dynamics system in which the Navier-Stokes equations are numerically evaluated. In EML, the sample surface is approximated to be a stationary boundary on which fluids are allowed to slip tangentially. Current models evaluate the system using a 2D axisymmetric mesh, allowing for reduced computation time and enforcing symmetry conditions along the internal boundary. The magnetohydrodynamic models use the forces applied to the sample by the magnetic field to calculate the induced flow within the sample. The flow pattern plotted in Fig. 1 shows the flow on the right-hand side, which results from the force distribution given on the left-hand side.

### Recent achievements

In the following, some highlights of completed experimental and modelling work related to various teams/projects are presented. The full list of team members of the projects can be found in the supplemental material.

### THERMOPROP/THERMOLAB

In the THERMOPROP/THERMOLAB projects, a large set of thermophysical property data was collected using the electromagnetic levitator ISS-EML. Refs. ^31,59^ also give an overview.

The measurements on board the ISS can be seen as benchmark experiments, since they are performed without the systematic error sources gravity and container. Since the surface tension and viscosity are measured by the oscillating drop method^40^, and evaluated by the Rayleigh^60^ and Lamb^61^ equations, the uncertainty of the measured surface oscillation frequency and damping time constant dominate the overall uncertainty. Typically, the surface oscillation frequency can be determined with an uncertainty better than ±0.25%. An additional uncertainty comes from the sample mass, which can be determined pre- and post-flight (mass loss during processing is negligible) with an accuracy of about ±0.1%. Hence, surface tension is usually obtained with an accuracy of about ±0.6%.

Regarding the viscosity, the damping time constant of the surface oscillations can usually be obtained with an accuracy of ±1%. Evaluation of the viscosity requires in addition knowledge of the sample radius, which can be obtained with an accuracy of ±0.8% respectively. This leads to a precision of the viscosity of about ±1.9%.

The specific heat capacity is determined by non-contact inductive modulation calorimetry^23,32^. Its accuracy relies on the accurate determination of the temperature oscillation amplitude and the amplitude of modulated power dissipation. The temperature can be measured with an accuracy of about ±0.1 K^22^, which translates to an accuracy of about ±1% in the determination of the temperature modulation amplitude. The power dissipation, calculated from the electrical resistivity and coil geometry is obtained with an accuracy mainly determined by the accuracy of the electrical measurement of the samples resistivity and radius. This is about ±0.05%^41^ for the electrical resistivity. The accuracy of the sample radius is similarly good. However, during measurement, the sample is typically heated and as such is slightly distorted. There, the accuracy of the overall measurement can be improved by the measurement of the sample diameter – leading to an effective sample diameter with an uncertainty in the order of the accuracy which is obtained by the optical method to determine the sample radius of about ±0.05%. The sensitivity of the power dissipation on the specific resistivity is about 10^−6^ W·(µΩ·cm)^−1^ and about 10^−3^ W·mm^−1^ for the sample radius. That way, the achievable accuracy of specific heat can be estimated to be all better than ±2%. In the following, a few highlights are presented. It must be mentioned, that for the ac calorimetry method, no a-priori knowledge of the total hemispherical emissivity is necessary^32^.

The complete set of thermophysical properties, namely surface tension, viscosity, heat capacity, total hemispherical emissivity, specific resistivity, and mass density was determined for the three Ni-based superalloys LEK94, MC2, and CMSX-10^62^.

**Fig. 2:**
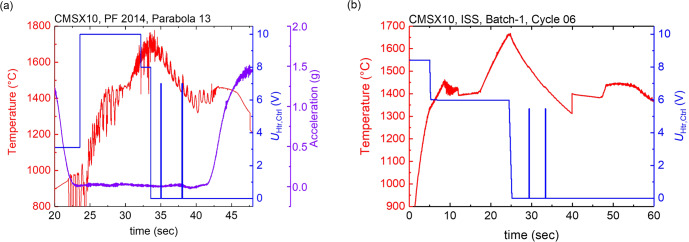
typical melt cycle for CMSX-10. **a** on a parabolic flight, ~10 s in the liquid phase (**b**) in the ISS-EML on board the ISS, about 30 s. in the liquid phase. Reproduced under the terms of the CC BY 4.0 license^31^. Copyright 2020, Wiley.

The reduced microgravity disturbances, as well as the longer available processing times on board the ISS have shown to reduce systematic and random errors introduced by the much smaller movement of the sample. This is most obvious, when comparing the temperature readings taken by a pyrometer during processing in a parabolic flight and on board the ISS. Fig. 2(a) shows the temperature time diagram of the Ni-based superalloy CMSX-10 processed in the parabolic flight, and Fig. 2(b) shows the same sample processed on board the ISS.

**Fig. 3:**
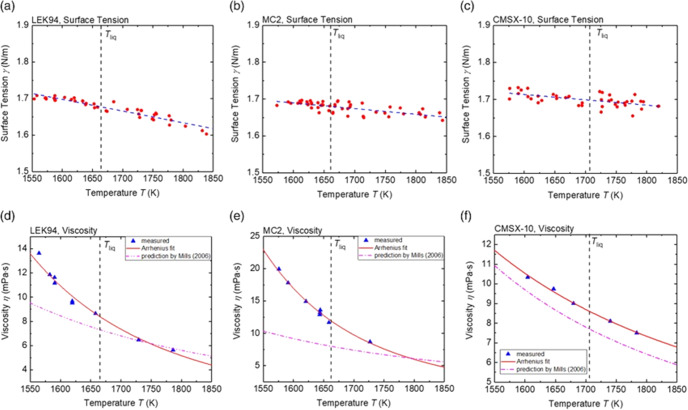
Surface tension and viscosity of three Ni-based superalloys. **a** surface tension of LEK94 (**b**) surface tension of MC2 (**c**) surface tension of CMSX-10 (**d**) viscosity of LEK94, (**e**) viscosity of MC2, (**f**) viscosity of CMSX-10. Dashed-dotted lines in (**d**), (**e**), (**f**) are model-curves after^85^. Reproduced under the terms of the CC BY 4.0 license^62^. Copyright 2020, Wiley.

The surface tension and viscosity of the three alloys LEK94, MC2, and CMSX-10 are shown in Fig. 3.

It is worth noting that the measurements show deviations from the corresponding theoretical data obtained by simple model-approaches, such as the empirical rule for the viscosities, reported by Mills et al^62^. This encourages the development of improved models.

A complete overview over the thermophysical properties of the Ni-based superalloys investigated in the course of the project can be found in ref. ^63^.

**Fig. 4:**
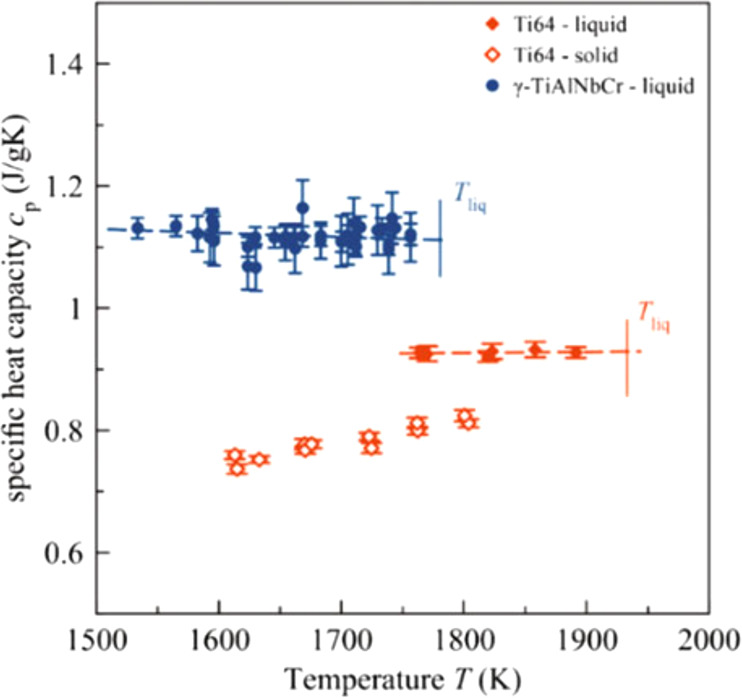
Specific heat of two Ti-based alloys. Specific heat of Ti64 in the solid and undercooled liquid phase, as well as for γ-Ti_48_Al_48_Nb_2_Cr_2_ in the undercooled liquid state. Reprinted with permission from Springer Nature^59^.

Other examples of high-temperature alloys investigated in the ISS-EML in the past were the Ti-Al alloys Ti64 (Ti-6Al-4V) and Ti48Al48Nb2Cr2. For both alloys, the full set of thermophysical properties was obtained. Exemplarily, Fig. 4 shows the specific heat capacity measured by the ac calorimetry method, obtained for both alloys^64,65^.

On ground, it was not possible to obtain the specific heat capacity in the undercooled liquid phase of the two rather reactive melts of Ti64 and Ti_46_Al_46_Nb_2_Cr_2_.

After the last solidification cycle of the Ti64 sample on board the ISS the sample has been investigated by SEM, 2D XRD and X-CT analysis to investigate structural specificities such as surface topography, elemental composition, crystalline phases and morphology. In that last cycle, the sample was undercooled 200 K below the liquidus temperature. On ground, such high undercoolings might not occur during casting, but maybe locally during 3D printing or welding.

**Fig. 5:**
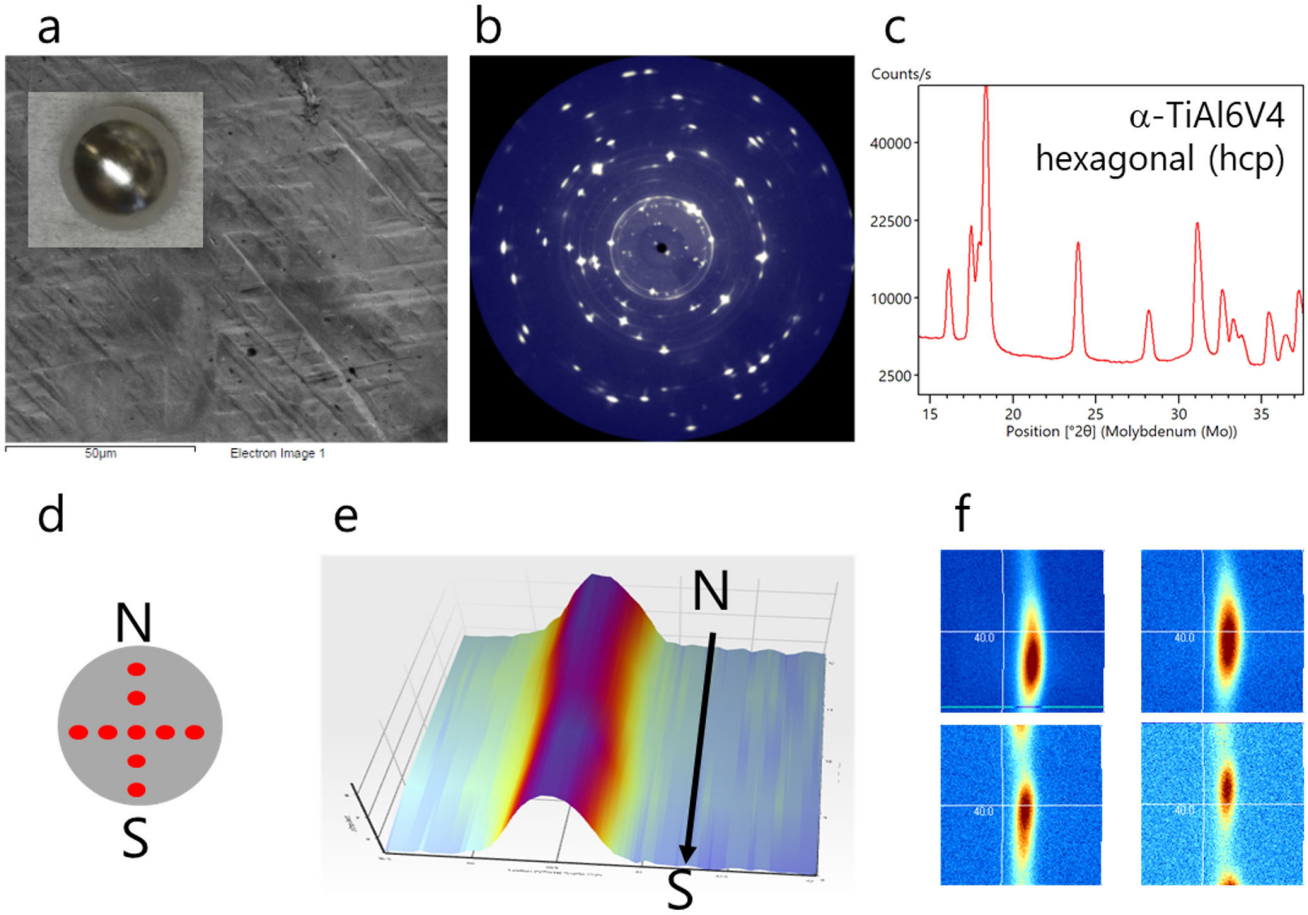
Analysis of the ISS processed sample Ti64. **a** SEM of sample surface (inset shows an optical image of the 6 mm ∅ sample specimen), (**b**) 2D XRD pattern measured in transmission mode, (**c**) 1D XRD pattern from sample center showing the Ti64 hexagonal (alpha) phase, (**d**) schematic of the sample (disc with a 6 mm ∅) with measurement points labeled in red, (**e**) 3D view on the 101 reflection monitored from the north (N) to the south (S) of the sample (x = 2Theta, Y = on-sample position, color code = intensity), (**f**) reciprocal space maps of the 101 reflection at different sample positions.

The SEM/EDX results indicate that the surface of the sphere appears smooth, shiny and almost defect free. Surface topography shows a pattern with elongated rectangular tiling (Fig. 5a). The elemental Ti-V-Al distribution is homogenous and corresponds to the nominal elemental composition.

The sample is highly crystalline with large crystallites. 2D XRD patterns of the surface and the cross section have been taken in reflection and transmission (Fig. 5b), respectively. The radial integration over 360 degrees results in a 1D diffraction pattern which can be indexed with the hexagonal α-phase (hcp, space group P6_3_/mmc).

A schematic of the sample (disc with a 6 mm ∅) is given in Fig. 5d showing the sample areas being investigated. A 3D view on the 101 reflection monitored from the north to the south of the sample (Fig. 5e) is shown together with selected reciprocal space maps of the same 101 reflection at different sample positions. Our study shows no major crystallographic differences from the out- to the inside of the ISS-processed sample.

**Fig. 6:**
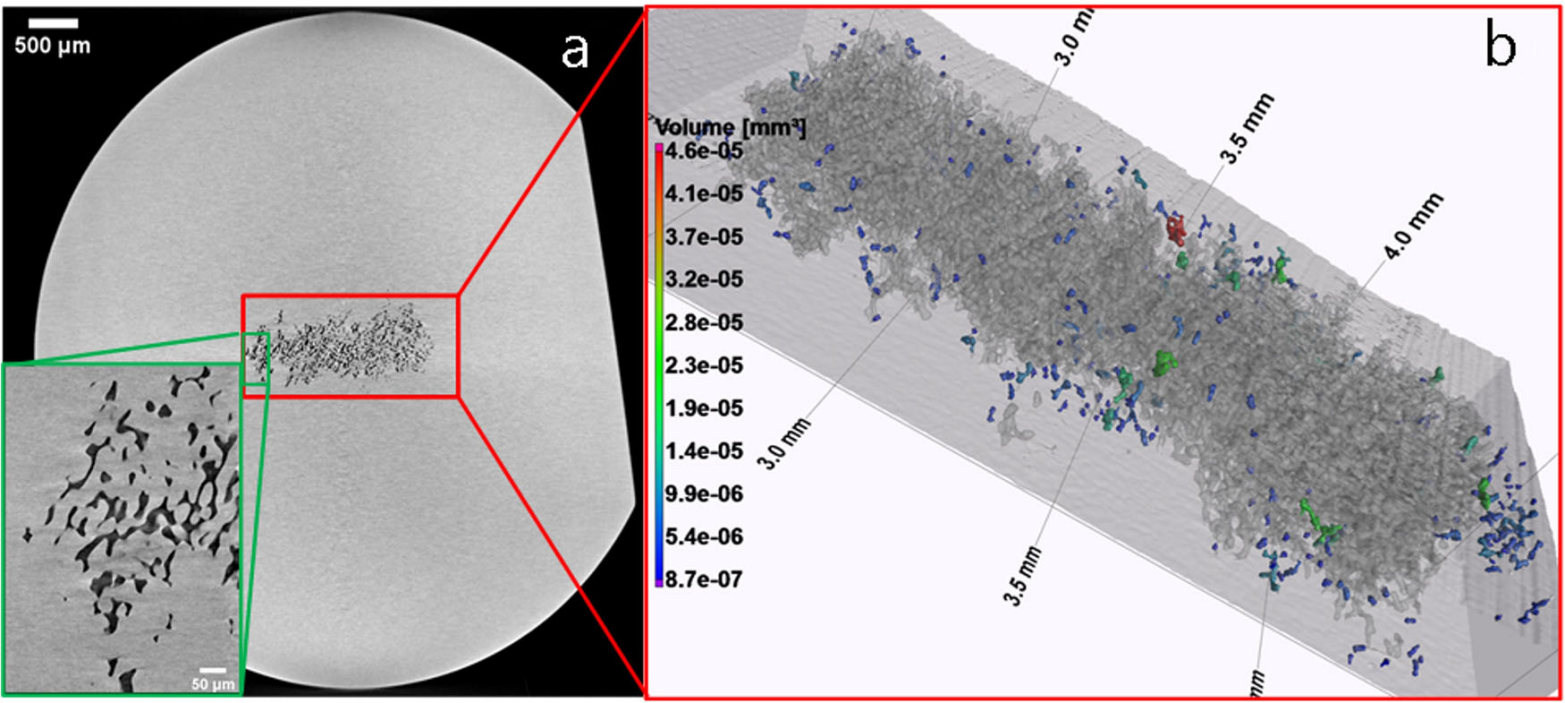
X-ray CT analysis. **a** CT slice showing voids in the center of the Ti64 sphere the inset in green shows an enlarged view and (**b**) 3D rendering of the central porous region enlarged showing large open porosity cloud interconnected to the surface (in gray) and smaller closed porosity (color scaled with the pore volume) not connected to the surface.

However, differences can be monitored investigating the morphology of the entire sample volume. An X-ray micro-CT volume analysis has been performed showing voids and porosity concentrated in the center of the sphere of 6 mm ∅ (Fig. 6). Outside of this area, the sample is very homogeneous and free of voids and defects down to the resolution level of the micro-CT, ca 1.5 µm.

The large void cloud in the center region of the sample mostly contains a large, inter-connected open pore structure, 11.85% of the volume in the red region of interest (ROI) shown in Fig. 6 and a small fraction of closed pores, 0.25% of the ROI volume. The mean equivalent pore size for the closed porosity is 17.5 µm.

**Fig. 7:**
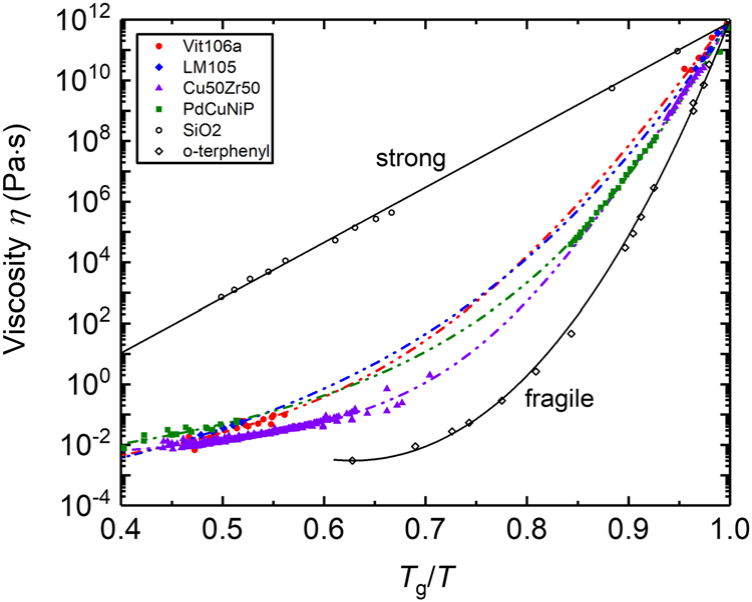
Viscosity of four metallic glasses. Viscosity of four metallic glasses are shown in the Angell plot over a wide temperature range. The high-temperature data (< 0.75 *T*_g_/*T*) was measured in microgravity^31,50,52,53^, while the data close to *T*_g_ has been measured on ground^86–89^. Also literature data of SiO_2_ and o-terphenyl^90^ obtained in ground-based measurements are shown for comparison. Reprinted with permission from Springer Nature^59^.

For metallic glasses, the viscosity is an important property. Dependent on the steepness of the viscosity increase around the glass transition temperature *T*_g_, during cooling, the glass forming melts are classified into strong and fragile liquids. While the viscosity of strong liquids shows an Arrhenius-type behavior, the viscosity of fragile liquids increases significantly around *T*_g_. Since the temperature dependence of viscosity reflects the change of the liquids kinetics during cooling, it is especially important to know the viscosity not only around *T*_g_, but also in the stable and undercooled liquid range. Fig. 7 shows an Angell plot of four metallic glass formers. The shown data is the combination of viscosity data obtained in the high-temperature stable and undercooled liquid phase on board the ISS and in several parabolic flight campaigns. Complementary data from literature, obtained on ground in the temperature range close to *T*_g_ is also shown. Data of similar quality have been obtained in pure ground-based ESL experiments^28,29,43,44^, where microgravity data partially served as benchmark.

**Fig. 8:**
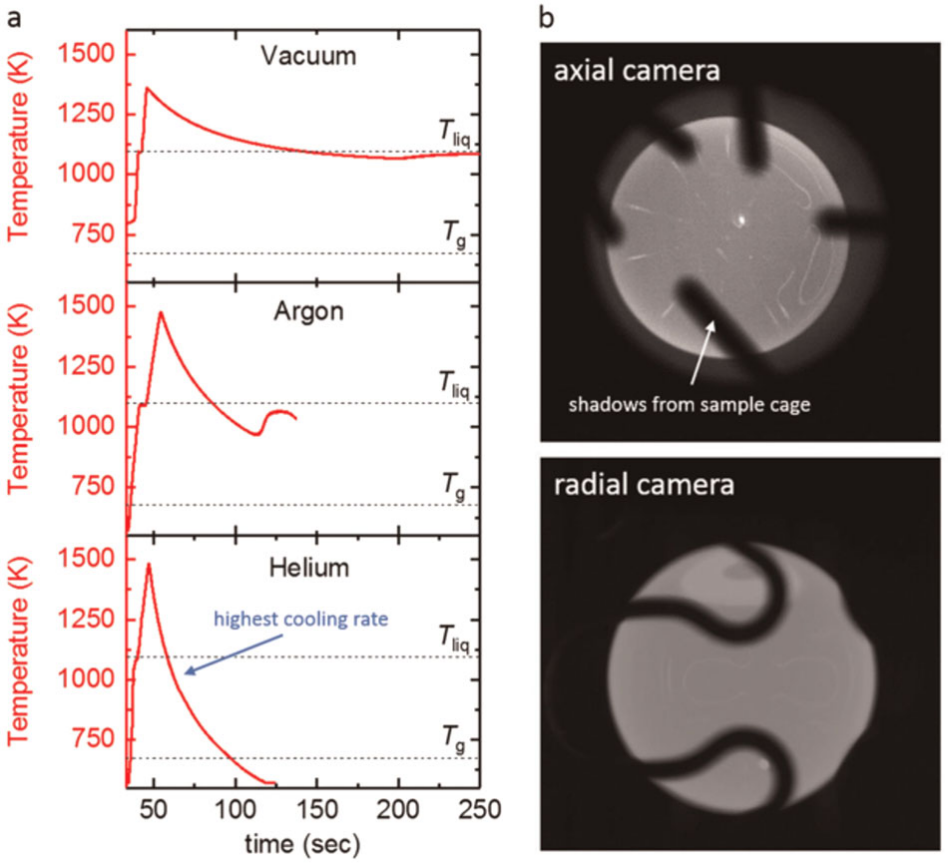
LM105 processed in the ISS-EML. **a** Temperature-time profiles of melt cycles performed in different atmospheres. **b** axial and radial camera images, showing the round molten sample. Shadows on the samples stem from the (non-contact) sample cage. Reproduced under the terms of the CC BY 4.0 license^50^. Copyright 2019, Springer Nature.

Since the electromagnetic levitator ISS-EML allows processing under the different process conditions that include Argon and Helium atmospheres, as well as Vacuum, three different cooling rates can be achieved. Fig. 8 shows typical temperature-time diagrams for the 6.5 mm diameter sphere of the alloy LM105 (Zr_52.5_Cu_17.9_Ni_14.6_Al_10_Ti_5_), recorded during melting and cooling in these three environments. The black shadows seen on samples stem from the wires of the (non-touching) sample cage that surrounds the sample. These shadows are not moving during the experiment, and hence, they could be easily excluded during image analysis (e.g. for the surface tension or optical density measurements). Since the cage wires only obscure a small portion of the samples edges, they do not contribute significantly to the measurement errors. The sample was molten, overheated and subsequently cooled freely. Cooling in vacuum is the slowest, since only heat loss by radiation takes place. Cooling in Argon and Helium involves both, radiation and heat conduction by the gas. The cooling rate in He atmosphere was about 8 K/s, very close to the reported critical cooling rate of LM105 of about 10 K/s^66^. No recalescence could be detected during cooling in He.

Furthermore, measurements of the electrical resistivity performed during cooling also confirm the vitrification of the sample during cooling in He^50^. Hence, to the best of our knowledge, this was the first production of a metallic glass sphere in space!

### ELFSTONE/LODESTARS

In a collaboration with the THERMOPROP/THERMOLAB group, an investigation was launched to investigate the influence of convection on steel alloy transformation kinetics and phase selection^67^. In this series of investigations, the unique attributes afforded by containerless ISS-EML processing^68,69^ are leveraged to select and control convection over a wide accessible range of conditions – from laminar to turbulent^70–72^. During thermophysical property measurements required to enable magnetohydrodynamic modeling of test conditions, it was found that convection introduces significant bias to measured results. It was evident that to obtain the desired measurement precision, the bias needed further investigation.

**Fig. 9:**
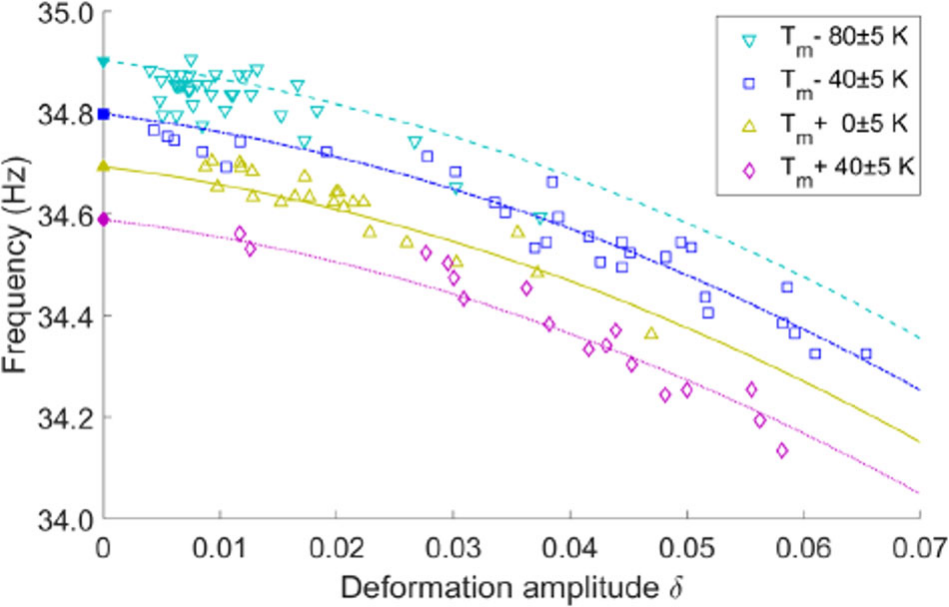
Observed frequency shift. Observed frequency shift at several test temperatures for a sample of nickel-based LEK-94 superalloy processed by the ThermoLab group using the ISS-EML facility. Reproduced from^73^, with permission from AIP Publishing.

Based on the strength of the excitation impulse amplitude, the surface deforms from a spherical shape to a prolate ellipsoid which then oscillates from prolate to oblate until the motion stops due to viscous damping. The oscillation frequency observed after pulse excitation is used to define the surface tension using the Rayleigh equation. The deformation that is induced is quantified using a deformation parameter *δ* which represents the change in instantaneous width *R*_w_ based on the original spherical radius *R*_o_ such that *R*_w_*/R*_o_ = *(1* ± *δ)*^73^. As seen in Fig. 9, for a series of test temperatures a negative parabolic deviation in frequency is observed. When normalized, this deviation collapses to a single simple parabolic plot to allow the impact of deformation to be eliminated.

**Fig. 10:**
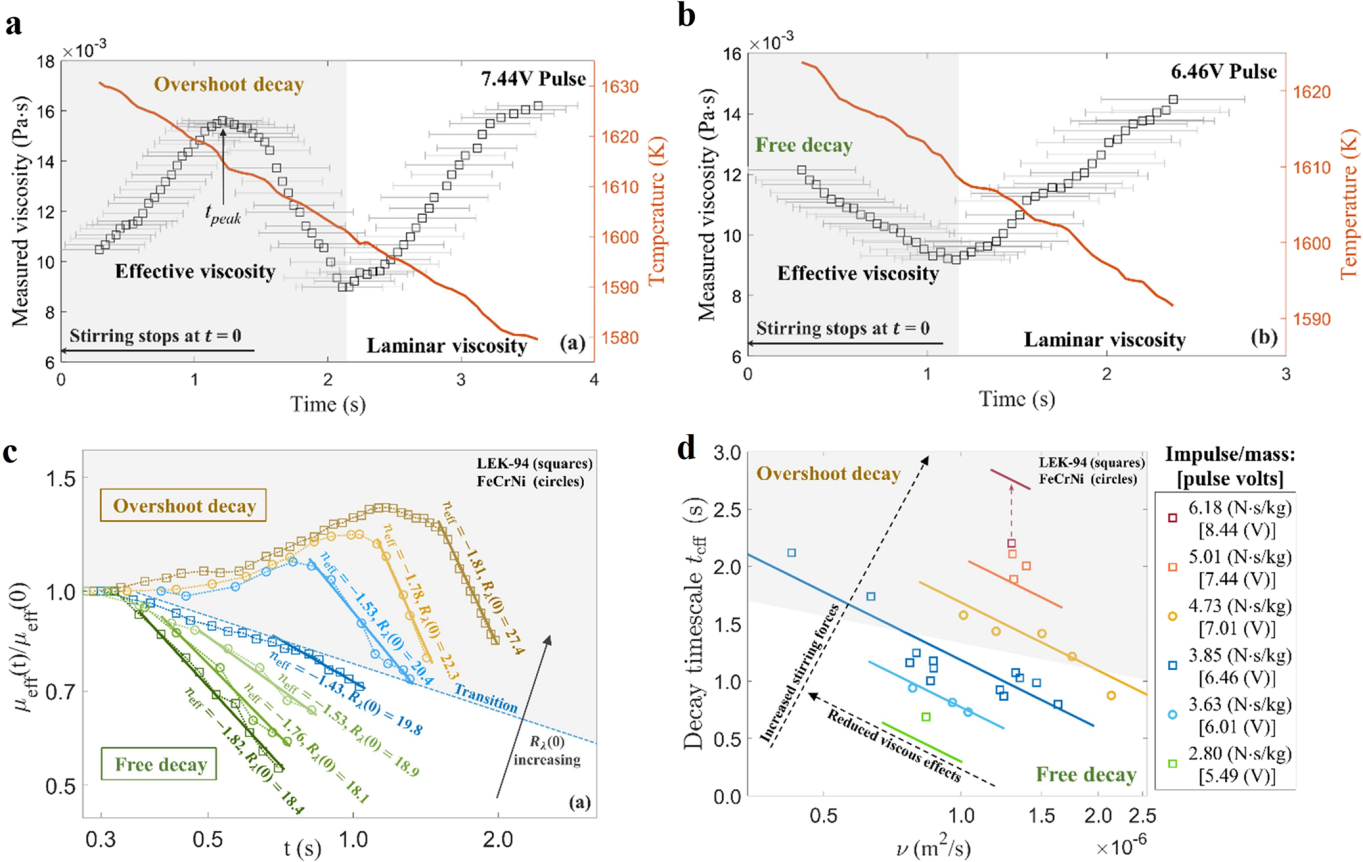
Measured apparent viscosity as a function of time. **a** for a strong excitation impulse exhibiting overshoot decay behavior pattern, and (**b**) for a moderately low excitation impulse exhibiting free decay behavior pattern, both as a function of relative time after pulse application and superimposed on the decreasing temperature profile. **c** Normalized viscosity as a function of time at various Taylor–Reynold’s numbers showing the overshoot and free decay pattern transition. **d** Critical impulse limitation to avoid overshoot delay and the timescale of the decay period. Reprinted with permission from Springer Nature^74^.

Similarly, the excitation impulse amplitude influences the oscillation decay that is used to quantify the fluid viscosity based on Lamb’s theory. Two regimes are observed following excitation as a sample is cooled^74^. We would expect the viscosity to rise during cooling but at high initial impulse the viscosity appears to rise, then fall, then rise again. This transition is shown in Fig. 10(a) and is known as overshoot decay and represents an initial measure of apparent turbulent viscosity immediately after excitation when internal flow induced by the pulse dominates. Immediately following this transient the viscosity drops to true values when laminar conditions are reestablished. Then it rises as the fluid cools. The other regime is known as free decay and here the impulse amplitude is smaller such that no turbulent regime is observed, and the apparent viscosity only falls and then rises when laminar conditions are reestablished as the temperature falls. The transition between overshoot and free decay is seen to occur at a Taylor–Reynold’s number ${R}_{\lambda }=\sqrt{20\rho {K}^{2}/3\mu \varepsilon }=20$ as seen in Fig. 10 (c). In the Figure, since temperature is falling, the apparent viscosity is a strong function of time and this *μ*_eff_*(t)* is normalized with the initial apparent viscosity at time zero *μ*_eff_*(0)* to highlight that the relative similarity in behavior pattern response for two different alloys – a nickel superalloy and a stainless steel.

In both the overshoot and free decay cases, the initial transient must be quantified such that a sufficient decay timescale is allowed to pass to avoid the apparent fall in viscosity associated with the decay of fluid flow initiated by the pulse. This is shown in Fig. 10(d) for the same two materials at different viscosities. The data set indicates that for ISS-EML testing an impulse of lower than 3.85 N‧s/kg, corresponding to a facility control voltage setting of 8.48 V, is required to avoid the overshoot decay regime and that the period where free decay extinction occurs extends from between 1–2 s over the range of kinematic viscosities tested.

An additional concern was identified during ground-based experiments on related ferrous alloys^75^ and thermophysical property testing of a selection of superalloy compositions^76^. Since testing was to be conducted on molten samples at elevated temperature over relatively long durations, preferential evaporation of volatile species from the melt was possible. In order to set limits on test conditions to ensure that the composition of the alloy does not shift during an experimental series, a method was developed to dynamically track mass loss for each element in solution based on its chemical activity. Results showed that under the conditions selected for testing using the ISS-EML facility there was no significant loss for any of the volatile species^77,78^.

These three examples show how systematic error could be quantified and then modelled to allow the researchers to correct errors introduced by deviation from the required small-deformation assumption during surface tension measurement, correct errors introduced by convection during measurement of viscosity, and quantify errors introduced by preferential evaporation during density or pulse oscillation evaluation.

Note that these investigations require accurate temperature measurement as the element-specific vapor pressure, activity, and density must be known. To this end, errors in pyrometry were investigated using the Guide to the expression of Uncertainty in Measurement (GUM) technique^79^ and found to contribute errors in temperature on the order of 12.4 K composed of two main components: an error of 11.4 K due to calibration at the known reference temperature and an error of 6.8 K due to potential changes in emissivity, view factor and transmissivity of the optical path for a representative Inconel superalloy sample. To complete the quantitative example, when evaluating overall uncertainty in density the errors are a combination of errors in temperature of 0.7%, in mass of 0.4%, in videographic radius measurement of 0.6%, and in volume of 1.7%. Noting that in using GUM the errors are not a simple sum of each individual contribution but rather an organized, synthesized result based on the relative interaction of each component, for this example errors in the calculation of temperature and density are orthogonal and these errors combine to yield an overall uncertainty in density of 1.8%.

### USTIP

Project USTIP was initiated to bring in magnetohydrodynamic models to support other experiments on ISS-EML. These models have been applied to a wide range of experiments on topics including nucleation^80,81^, thermophysical property measurements^82^, and forthcoming experiments on the transition to turbulent flow in EML drops. The models rely on the calculated magnetic field to determine the resulting magnitude and pattern of the flow. Fig. 1 shows a “heater-dominated flow pattern” in which the largest forces are exerted by the dipole field used to heat the sample and induce surface oscillations. The forces in a heater-dominated case are largest along the surface of the sample along the equator. This gives a flow pattern with two recirculating loops in which the flow is driven into the sample near the equator and returns to the surface of the sample along the poles. These models have been used to estimate the shear-strain rate to calculated the probability of interactions between subcritical nuclei in a wide range of materials including Vit106, Ti_39.5_Zr_39.5_Ni_21_, Zr_64_Ni_36_, and Cu_50_Zr_50_^81^. Other recent applications of these models include evaluating the flow for turbulent conditions during oscillating drop measurements in germanium^82^ and Fe_10_Si.

Recent work by USTIP has extended the magnetohydrodynamic flow to encompass transient flows during EML experiments. These were developed to further study the occurrence of dynamic nucleation in zirconium^80^ and zirconium nickel^83^. These models use a commercial computational fluid dynamics package with magnetic forces calculated by the method of mutual inductances. Details of the model, including properties and boundary conditions used, are given in^83^.

The transient models have also been used to investigate the effects of an excitation pulse on the internal flow of the drop. The excitation pulse consists of a sharp increase in the heating field for a short duration, typically about 100 ms. This pulse excites surface oscillations in the sample, as mentioned above, and also sometimes triggers solidification. It is believed that the mechanism for triggering solidification is dynamic nucleation.

**Fig. 11:**
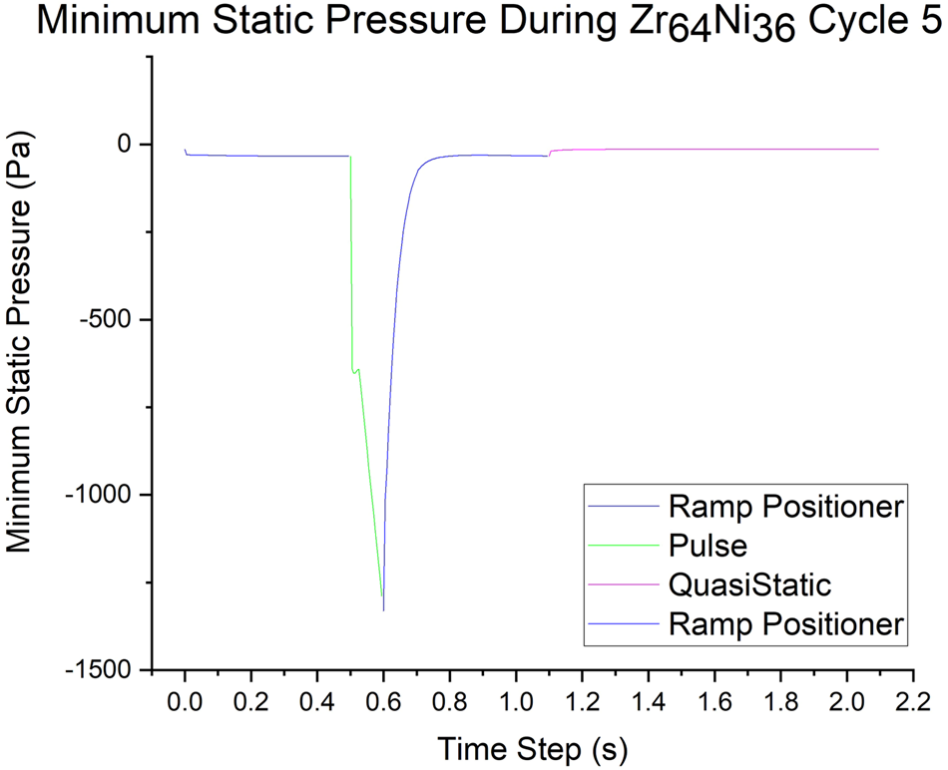
The maximum hydrostatic tension. The maximum hydrostatic tension (negative hydrostatic pressure) is plotted over the range of the excitation pulse calculated for dynamic nucleation experiments in the ISS-EML using a Zr_64_Ni_36_ sample.

In dynamic nucleation, the flow is theorized to initiate solidification through the interaction of voids in the liquid with local pressure variations resulting from the flow. The transient models allowed the effects of pressure and velocity within the drop to be calculated over the range of the excitation pulses observed to trigger nucleation in Z_64_Ni_34_. The hydrostatic tension in the sample is plotted in Fig. 11.

In the pulsed dynamic nucleation experiments, the models show that the flow is initially quite low, even as the positioner is increased prior to the heater pulse. The circulating flow does result in a small hydrostatic tension due to the curvature of the flow pattern. Then the heater pulse is applied. The pulse causes rapid acceleration of the flow, resulting in a hydrostatic tension of approximately 1300 Pa (Fig. 11, green line). After the pulse is complete, the flow quickly relaxes, and the hydrostatic tension returns to the steady-state value near zero. (Fig. 11, blue line). Current work is on-going applying the transient-magnetohydrodynamic models to additional experimental conditions requiring an in-depth understanding of time-dependent flow effects on experiments in EML.

### Outlook and summary

A number of industries have a strong need for technological innovations, such as in information technology, aerospace, automotive, bio-medical, and material manufacturing. At the same time, they need to achieve high-quality, high value-added products, and environmental sustainability. In the future, further improvements of modern industrial processes, implementation of new manufacturing processes, based on simulations and the precise knowledge of thermophysical properties will become increasingly important. The description of solidification physics spans over a large number of length-scales, from atomistic models on the angstrom and nm scale up to macroscopic models of process simulations on the meter scale. In order to gain new insights, future simulations must bridge the gap between these length-scales.

As the ESA SciSpace White Paper #06: Materials Science presents^6^, future experiments will be a continuation of the ongoing and concluded activities, exploring additional structural, functional, and high-performance materials, as well as novel materials. An example of such novel materials are e.g. sulphur-containing bulk metallic glasses that will be investigated by the project BMW.

Additionally, emerging topics of materials science, related to space exploration, such as in-space manufacturing and in-situ resource utilization will generate new need for the characterization of molten metallic materials and their solidification in microgravity.

Furthermore, future experiments will increase the knowledge about basic scientific questions on liquid matter. This could lead to better understanding of several critical phenomena that can take place during solidification, such as glass formation.

Solidification is also a surface- and interface-driven process. Thus, in order to get a full understanding, the role of surface-active minority species, most importantly Oxygen and Sulphur, need to be clarified. To this end, the OXYTHERM project envisages to install and use an oxygen control sensor (OCS) at the EML-ISS. Monitoring and control of the oxygen partial pressure allows the study of adsorption/desorption processes of oxygen and their influence on the thermophysical properties. In addition, the OCS will assure even more well defined conditions which lead to further improvement of the yet precise results.

By the combination of precise experiments with atomic simulations, one could access information on the liquids inner interactions that are otherwise inaccessible. Simulation validation may be obtained by the comparison of macroscopic material properties, such as, viscosity, diffusivity, density, surface tension, specific heat, electrical- and thermal conductivity, etc., extracted by the atomistic modelling and precision measurements performed under microgravity. Pioneering investigations of this kind have been started already, and will be continued in the future^54,55,84^.

## Supplementary information

Supplemental Material

## Data Availability

The datasets generated during and/or analysed during the current study are available from the corresponding author on reasonable request.
