# Supplementary material for: Electromagnetic levitation containerless processing of metallic materials in microgravity: thermophysical properties
Source: NPJ Microgravity. 2023 May 2;9:34. doi: 10.1038/s41526-023-00281-4 (PMC10154313; doi:10.1038/s41526-023-00281-4)
Supplement: Supplementary file 1 — Supplemental Material [file 41526_2023_281_MOESM1_ESM.pdf]

SUPPLEMENTAL MATERIAL

Materials Science Coordinator at ESA:

European Space Agency (ESA), the Netherlands,
W. Sillekens
European Space Research and Technology Centre (ESTEC), Keplerlaan 1, Postbus 299, 2200 AG Noordwijk, the Netherlands

List of participants in the involved project consortia:

THERMOPROP - Thermophysical properties of liquid metals for industrial process design

Ulm University, Germany
H.-J. Fecht (Team Coordinator), M. Mohr, R. Wunderlich, Y. Dong,
Ulm University, Institute of Functional Nanosystems, Albert-Einstein-Allee 47, 89081 Ulm, Germany

DLR Cologne, Germany
J. Brillo
DLR German Aerospace Center, Institute of Materials Physics in Space, 51147 Köln, Germany

CNRS-SIMAP, France
Y. Champion, O. Budenkova, A. Fournier-Gagnoud
Centre National de la Recherche Scientifique, CNRS-SIMAP, 1130 rue de la Piscine, 38402 St. Martin D'Heres, France

CNR-ICMATE Genova, Italy
R. Novakovic, E. Ricci
CNR-ICMATE Dept. of Genova, Via de Marini, 6, 16149 Genova, Italy

Università di Torino, Italy
L. Battezzati,
Dipartimento di Chimica Inorganica, Chimica Fisica e Chimica dei Materiali, Via Pietro Giuria, 7, 10125 Torino, Italy

Empa, Switzerland
A. Dommann, A. Neels
Empa, Lerchenfeldstr. 5, CH-9014 St. Gallen, Switzerland

University of Alberta, Canada
H. Henein,
Dept. of Chemical & Materials Engineering, University of Alberta, Edmonton, AB T6G 2G6, Canada

University of Warwick, United Kingdom
P. Srirangam,
WMG, International Digital Laboratory, University of Warwick, Coventry CV4 7AL, United Kingdom

THERMOLAB - Thermophysical properties of liquid metallic alloys – modeling of industrial solidification processes and development of advanced products

Ulm University, Germany
H.-J. Fecht (Team Coordinator), M. Mohr, R. Wunderlich, Y. Dong
Ulm University, Institute of Functional Nanosystems, Albert-Einstein-Allee 47, 89081 Ulm, Germany

DLR Cologne, Germany
J. Brillo
DLR German Aerospace Center, Institute of Materials Physics in Space, 51147 Köln, Germany

CNRS-SIMAP, France
Y. Champion, O. Budenkova, A. Fournier-Gagnoud
Centre National de la Recherche Scientifique, CNRS-SIMAP, 1130 rue de la Piscine, 38402 St. Martin D'Heres, France

CNR-ICMATE Genova, Italy
R. Novakovic, E. Ricci
CNR-ICMATE Dept. of Genova, Via de Marini, 6, 16149 Genova, Italy

Università di Torino, Italy
L. Battezzati,
Dipartimento di Chimica Inorganica, Chimica Fisica e Chimica dei Materiali, Via Pietro Giuria, 7, 10125 Torino, Italy

Empa, Switzerland
A. Dommann, A. Neels,
Empa, Lerchenfeldstr. 5, CH-9014 St. Gallen, Switzerland

University of Alberta, Canada
H. Henein,
Dept. of Chemical & Materials Engineering, University of Alberta, Edmonton, AB T6G 2G6, Canada

University of Warwick, United Kingdom
P. Srirangam,
WMG, International Digital Laboratory, University of Warwick, Coventry CV4 7AL, United Kingdom

Tufts University, USA
D. M. Matson
Tufts University, Department of Mechanical Engineering, 200 College Ave., Medford, MA 02155, USA

University of Massachusetts, USA
R. Hyers, G. P. Bracker
University of Massachusetts, Department of Mechanical an Industrial Engineering, 160 Governors Drive, Amherst, MA 01003-2210, USA

Washington University, USA
K. Kelton, A. Gangopadhyay
Washington University, Department of Physics, 1 Brookings Drive – Campus Box 1105, St. Louis, MO 63130, USA

NASA Jet Propulsion Laboratory, USA
D. Hofmann,
Engineering & Science Directorate, NASA Jet Propulsion Laboratory/California Institute of Technology, MS 18-105, 4800 Oak Grove Dr., Pasadena, CA 91109, USA

Zhejiang University, P. R. China
J. Z. Jiang,
Zhejiang University, International Center for New-Structured Materials, Department of Materials Science and Engineering, 310027 Hangzhou, P. R. China

Gakushuin University, Japan
M. Watanabe,
Gakushuin University, Department of Physics, 1-5-1 Mejiro, Toshima, Tokyo 171-8588, Japan

Japan Aerospace Exploration Agency (JAXA), Japan
T. Ishikawa,
Japan Aerospace Exploration Agency (JAXA), 2-1-2 Sengen, Tsukuba 305-8505, Japan

Keio University, Japan
T. Hibiya,
Keio University, Graduate School of System Design and Management, 1-1 Hiyoshi 4-chome, Kohoku-u Yokohama 223-8526, Japan

Osaka University, Japan
T. Tanaka,
Osaka University, Division of Materials and Manufacturing Science, Graduate School of Engineering, 2-1 Yamadaoka, Suita, Osaka 565-0871, Japan

Tohoku University, Japan
H. Fukuyama,
Tohoku University, Institute of Multidisciplinary Research for Advanced Materials, 2-1-1 Katahira, Aoba-ku, Sendai 980-8577, Japan

Indian Institute of Technology Kharagpur, India
I. Manna,
Indian Institute of Technology Kharagpur, Department of Metallurgical and Materials Engineering
IIT, Kharagpur, West Bengal – 721 3012, India

Korea Research Institute of Standards and Science, South Korea
G. W. Lee,
Heat & Light Metrology Group, Division of Physical Metrology, Korea Research Institute of Standards and Science, 209 Gajeong-Ro, Yusseong-Gu, Daejeon 305-340, South Korea

Chinese Academy of Science, China
B. Wei,
Chinese Academy of Science, Vice president of Northwestern Polytechnical University, Space Materials Science and Technology, P. R. China

ELFSTONE/LODESTAR - Electromagnetic levitation flight support for transient observation of nucleation events / Levitation observation of dendrite evolution in steel ternary alloy rapid solidification

Tufts University, USA
D. M. Matson
Tufts University, Department of Mechanical Engineering, 200 College Ave., Medford, MA 02155, USA

USTIP - Unified support for THERMOLAB, ICOPROSOL, and PARSEC
(ICOPROSOL: Thermophysical properties and solidification behavior of undercooled Ti-Zr-Ni liquids showing an icosahedral shortrange)
(PARSEC: Peritectic alloy rapid solidification with electromagnetic convection order)

University of Massachusetts, USA
R. Hyers, G. P. Bracker
University of Massachusetts, Department of Mechanical an Industrial Engineering, 160 Governors Drive, Amherst, MA 01003-2210, USA

BMW
Saarland University, Germany
R. Busch, L. Ruschel
Saarland University, Chair of Metallic Materials, Campus C63, 66123 Saarbrücken, Germany

DLR Cologne, Germany
A. Meyer
DLR German Aerospace Center, Institute of Materials Physics in Space, 51147 Köln, Germany

University of Münster, Germany
G. Wilde
University of Münster, Institute of Materials Physics, 48149 Münster, Germany

OXYTHERM - Thermophysical properties of liquid alloys under oxygen influence

DLR Cologne, Germany
J. Brillo
DLR German Aerospace Center, Institute of Materials Physics in Space, 51147 Köln, Germany

Clausthal University of Technology, Germany
H. Fritze
Clausthal University of Technology, Institut für Energieforschung und Physikalische Technologien, 38640 Goslar, Germany

CNR-ICMATE Genova, Italy
R. Novakovic, E. Ricci, D. Giuranno
CNR-ICMATE Dept. of Genova, Via de Marini, 6, 16149 Genova, Italy

Doshisha University, Japan
H. Kobatake
Doshisha University, Organization for Research Initiatives & Development,
1-3 Tatara Miyakodani, Kyotanabe, Kyoto, 610-0394, Japan

Korea University, Korea
J. Lee
Korea University, Department of Materials and Science and Engineering, Anam-Dong, Seoul 136-713, Korea

University of Miskolc, Hungary
G. Kaptay
University of Miskolc, Department of Nano-composites, Miskolc, Egyetemvaros,
HU-3515 Hungary.

Chiba Institute of Technology, Japan
S. Ozawa
Chiba Institute of Technology (CIT), Department of Mechanical Science and Engineering,
2-17-1 Tsudanuma, Narashino, Chiba 275-0016, Japan.

Gakushuin University, Japan
M. Watanabe,
Gakushuin University, Department of Physics, 1-5-1 Mejiro, Toshima, Tokyo 171-8588, Japan

Tohoku University, Japan
H. Fukuyama
Institute of Multidisciplinary Research for Advanced Materials (IMRAM), Tohoku University, 2-1-1, Katahira, Aoba-ku, Sendai-shi, Miyagi, 980-8577, Japan
